# Supplementary material for: Pasteurella sp. associated with fatal septicaemia in six African elephants
Source: Nat Commun. 2023 Oct 25;14:6398. doi: 10.1038/s41467-023-41987-z (PMC10600241; doi:10.1038/s41467-023-41987-z)
Supplement: Supplementary file 1 — Supplementary Information [file 41467_2023_41987_MOESM1_ESM.pdf]

Supplementary Information

Pasteurella sp. associated with fatal septicaemia in six African elephants in Zimbabwe

Chris M. Foggin[1*], Laura E. Rosen[2*,†], Marijke M. Henton[3], Angela Buys[4], Toby Floyd[5], Andrew D. Turner[6], Jonathan Tarbin[7], Antony S. Lloyd[7], Columbas Chaitezvi[8], Richard J. Ellis[9], Helen C. Roberts[10], Akbar Dastjerdi[11], Alejandro Nunez[5], Arnoud H. M. van Vliet[12], Falko Steinbach[11,12]

[1] Victoria Falls Wildlife Trust, Victoria Falls, Zimbabwe
[2] Transboundary Epidemiology Analytics, LLC, Alpharetta, Georgia, USA
[3] Vetdiagnostix, Blue Hills, Midrand, South Africa
[4] Design Biologix, Erasmusrand, Pretoria, South Africa
[5] Pathology and Animal Sciences Department, Animal and Plant Health AgencyWeybridge, Addlestone, Surrey KT15 3NB, UK
[6] Centre for Environment Fisheries and Aquaculture Science, The Nothe, Weymouth, Dorset DT4 8UB, UK
[7] Fera Science, Biotech Campus, York YO41 1LZ, UK
[8] Zimbabwe Parks & Wildlife Management Authority, Harare, Zimbabwe
[9] Surveillance and Laboratory Services Department, Animal and Plant Health Agency Weybridge, Addlestone, Surrey KT15 3NB, UK
[10] Department for Environment Food & Rural Affairs, Nobel House, 17 Smith Square, London SW1P 3JR, UK
[11] Virology Department, Animal and Plant Health Agency Weybridge, Addlestone, Surrey KT15 3NB, UK
[12] Department of Comparative Biomedical Sciences, School of Veterinary Medicine, Faculty of Health and Medical Sciences, University of Surrey, Guildford GU2 7AL, UK
[*]These authors contributed equally to this work
[†] Present address: Victoria Falls Wildlife Trust, Victoria Falls, Zimbabwe

**Corresponding address: kazaepi@gmail.com

Supplementary Methods

Veterinary investigation

All samples were initially taken to the VFWT Laboratory (Victoria Falls, Zimbabwe), where they were either analysed or prepared for analysis at other laboratories. Blood smears were stained using standard Giemsa stain and examined with light microscopy. PCR for detection of anthrax DNA was performed according to a previously published method[1,2]. Briefly, DNA was extracted from blood, liver and/or spleen, from 14 elephants (QIAmp DNA mini kit, QIAGEN, Maryland, USA). The primer sequences were as follows: Ba813 R1 (TTAATTCACTTGCAACTGATGGG), Ba813 R2 (AACGATAGCTCCTACATTTGGAG), LEF3 (CTTTTGCATATTATATCGAGC), LEF4 (GAATCACGAATATCAATTTGTAGC), CAP57 (ACTCGTTTTTAATCAGCCCG), CAP58 (GGTAACCCTTGTCTTTGAAT). Master mix was prepared with 3 µl PCR grade water, 10 µl Platinum Hot Start 2x Master mix polymerase (Thermo Fisher Scientific catalogue number 13000012), 10 µM forward primers (0.25 µl R1, 0.25 µl CAP57, 0.5 µl LEF3), and 10 µM reverse primers (0.25 µl R2, 0.25 µl CAP58, 0.5 µl LEF4). The cycling conditions were as follows: denaturation at 94°C for 4 min, amplification using 35 cycles of 94°C for 40 sec, 57°C for 40 sec, and 72°C for 40 sec, and final extension at 72°C for 5 min. Samples were analysed by agarose gel electrophoresis with ethidium bromide.

Liver and spleen swabs in charcoal agar from two elephants were initially submitted to Zimvet Laboratory (Harare, Zimbabwe) for bacterial culture. Liver swabs from three elephants, together with swabs from the water source closest to the area where the majority of deaths occurred (‘Lion Pan’), were then submitted to VetDiagnostix Veterinary Pathology Services Pvt Ltd (Midrand, Republic of South Africa) for bacterial culture. Later, FTA cards prepared from blood and organ samples were submitted to Inqaba Biotechnical Industries (Pty) Ltd (Pretoria, Republic of South Africa). One liver and one lung swab, respectively from two elephants, together with two DNA extracts, formalin-fixed organ samples from two elephants plus water and sludge from ‘Lion Pan’ and stomach fluid from two elephants, were submitted to the Animal and Plant Health Agency (APHA; Weybridge), which

shared the samples for toxicology with the Centre for Environment, Fisheries and Aquaculture Science (CEFAS; Weymouth) and Fera Science Ltd (York, all UK) for further analysis.

Toxicological investigation

Stomach contents from two elephants (VF20/112 & 113) and pan-water sample and sediment samples from a waterhole (VF20/117) were sent for toxicological analysis at CEFAS and Fera respectively.

Targeted toxicological analysis: Samples were found to contain solid material, so were all initially centrifuged (4,500 g for 10 mins). Supernatants were filtered (0.2 µm) and processed as water samples. Remaining duplicate solid pellets were extracted for both aqueous and organic solvent-extractable toxins using 80% MeOH and 1% acetic acid extractions respectively.

Methanolic extraction prior to the analysis of microcystins was conducted using the method of Turner et al.[3]. In brief, solvent was added to solid pellets giving a 10:1 solvent:sample ratio, prior to a 3 min vortex mix and centrifugation. Water and pellet supernatants were subsequently subjected to microcystin analysis using reverse-phase ultra-high performance liquid chromatography with tandem mass spectrometry (UHPLC-MS/MS)[4]. Microcystin analogues incorporated within the method were MC-LR, RR, LA, LY, LF, LW, YR, WR, Asp3 MC-LR, HilR, HtyR, and D-Asp3 MC-RR, together with the additional cyanotoxin Nodularin (Nod).

Acidic extraction for analysis of saxitoxins, tetrodotoxins, anatoxin and cylindrospermopsin conducted according to a slightly modified version of Turner et al.[5]. 1% acetic acid was added to pellets to give a 2:1 solvent:sample ratio, prior to a 3 min vortex mix before being placed into a boiling water batch for 2 mins. After cooling under running water for 2 mins, extracts were centrifuged and the supernatants subjected to a desalting graphitic carbon-based solid phase extraction (SPE) as [6]. 100 µL of mixed SPE eluates were further diluted with 300 µL of acetonitrile prior to analysis. Water and pellet extracts were analysed using ultra-high performance hydrophilic interaction liquid chromatography (HILIC) with tandem mass spectrometry detection (HILIC-MS/MS) for detection of saxitoxin analogues (STXs), Tetrodotoxin (TTX)[7] together with anatoxin-a (ATX) and

cylindrospermopsin (CYN). STX analogues incorporated into the method included STX, dcSTX, NEO, dcNEO, doSTX, C1-4, GTX1-6 and dcGTX1-4. Additional analysis was conducted for detection of a larger range of TTX analogues (TTXs) as[4].

Analyte detection for both methods was conducted by running samples against calibration solutions containing known concentrations of toxin analytes, with quantitation achieved for any detected toxins using an external calibration (minimum six concentration levels). Quality controls were performed for by running samples in a batch containing positive and negative control samples, as well as instrumental blanks and spiked water samples. Limits of detection for each analyte range from 0.1 – 1.3 µg/L for microcystins and 0.4 – 13 µg saxitoxin equivalents (STX eq)/L for individual STXs and TTXs[4,7].

Non-targeted toxicological analysis: For stomach content and water analysis, two monotraps (DCC18 - octadecyl C18 + activated carbon and DSC18 – octadecyl C18) were added to the samples. The samples were shaken using a mechanical shaker for approximately 18 hours. The monotraps were then removed, dried, and back-extracted with 600µL acetonitrile. For LC-TOF-MS analysis, 150µL was removed and 27.5µL water added, followed by 15µL 1µg/mL internal standard mix in acetonitrile (Fraction B LC). For GC-QTOF-MS analysis, a second 150µL portion was removed (Fraction B GC). The remaining sample, after removal of the monotraps, was centrifuged (872 g, 10 minutes). 150µL of the supernatant was removed and 15µL 1µg/mL internal standard mix in acetonitrile added (Fraction C LC). The remaining supernatant was extracted with 600 (water) or 800 (stomach contents) µL dichloromethane. 150µL of the dichloromethane layer was removed for analysis (Fraction E GC). The residual solid after centrifugation was then re-extracted with 600µL acetonitrile by ultrasonication and centrifuged (2000 rpm, 10 min). For LC-TOF-MS analysis, 150µL was removed and 27.5µL water added, followed by 15µL 1µg/mL internal standard mix in acetonitrile (Fraction D LC). For GC-QTOF-MS analysis, a second 150µL portion was removed (Fraction D GC). The residual solid was then extracted with 600 µL dichloromethane by ultrasonication. 150µL of the dichloromethane layer was removed for analysis (Fraction F GC).

For sediment analysis, the sample was centrifuged (12100 g, 5 minutes) first. For LC-TOF-MS analysis, 150μL of the supernatant was removed and 15μL 1 μg/mL internal standard mix in acetonitrile added (Fraction A LC) added. The remaining supernatant was extracted with 600μL dichloromethane. 150μL of the dichloromethane layer was removed for analysis (Fraction E GC). The residual solid after centrifugation was then extracted with 600μL acetonitrile by ultrasonication and centrifugation (872 g, 10 min). For LC-TOF-MS analysis, 150μL was removed and 27.5μL water added, followed by the addition of 15μL 1μg/mL internal standard mix in acetonitrile (Fraction D LC). For GC-QTOF-MS analysis, a second 150μL portion was removed (Fraction D GC). The residual solid was then extracted with 600μL dichloromethane by ultrasonication. 150μL of the dichloromethane layer was removed for analysis (Fraction F GC).

Portions of the solvent extracts were analysed using an Agilent 7890B gas chromatograph coupled with an Agilent 7200 Q-TOF detector by splitless injection of 1 μL of the extract onto a DB-5MS UI capillary column (30 m x 0.25 mm i.d. x 0.25 μm film thickness). Following injection, the oven was held at 40 °C for 2 minutes and then raised at 10 °C/minute to 320 °C and held for 5 minutes. The inlet was held at 250 °C and helium (1 mL/minute constant flow) was employed as the carrier gas. The MS was operated in electron impact mode monitoring from 30 – 750 amu.

Portions of the solvent extracts were analysed using an Agilent 1290 infinity liquid chromatograph coupled with an Agilent 6530 QTOF Chromatographic separation was achieved on a Zorbax SB-Aq (50 x 2.1mm, 1.8 μm) held at 60ºC. The mobile phases comprised of water containing 0.2% acetic acid and methanol containing 0.2% acetic acid (B). The gradient started at 2% B and changed to 98% B at 13 minutes before returning to 2% B at 19 minutes. The flow rate was 0.6 mL/minute and the injection volume was 2 μL. The MS was operated in positive and negative mode electrospray with nebuliser pressure 45 psi, capillary voltage 4000 V (+ve) and 3500 V (-ve), nozzle voltage 1000 V, gas temperature 325 ºC, sheath gas temperature 350 ºC, drying gas flow 10 L/minute, sheath gas flow 11 L/minute, skimmer voltage 65 V, fragmentor voltage 140 V and octopole RF voltage 750 V. The mass range monitored was 50 – 1600 m/z at a scan rate of 1.5 spectra/second.

Data analysis of GC-QTOF-MS data was carried out using the MassHunter Unknowns Analysis B.0.9.00 software and matching deconvoluted spectra to the NIST library (match factor>70). In-house bespoke software was used to align and profile replicate data versus the appropriate solvent control.

Data analysis of LC-TOF-MS data was carried out using MassHunter Qualitative 10.0 software using the Find-By-Formula algorithm comparing to an in-house database of 2009 compounds consisting of biological toxins (fyco-, myco-, phyto- etc.) and pesticides (insecticides, herbicides, fungicides etc).

Core genome multilocus sequence typing (cgMLST)

A core genome MLST scheme was developed for *Pasteurella* spp. using chewBBACA version 2.8.5[8] using the tutorial (https://github.com/B-UMMI/chewBBACA_tutorial). A training file for *P. multocida* Pm70 (Accession number GCA_000006825.1) was generated using Prodigal version 2.6.3[9]. A whole genome MLST scheme (wgMLST) was initially generated using 15 *P. multocida* genomes and the VF20HR genome sequence, and contained 3,645 open reading frames (ORFs), which was subsequently reduced to 681 ORFs for the core genome scheme. The chewBBACA allele calls were used for generation of phylogenetic trees by using GrapeTree version 1.5.0 with the RapidNJ algorithm[10]. The *P. multocida* cgMLST scheme with allele sequences and training file is available from FigShare (https://doi.org/10.6084/m9.figshare.21791843.v1).

Genetic analysis for viruses

To determine the possible involvement of viral infections, two complementary approaches to the above were undertaken. First, the WGS DNA analysis was complemented by a templated and de novo search analysis for viruses such as described recently[11]. Further, DNA and RNA were extracted from the fixed tissue samples received at APHA using QIAamp DNA FFPE Tissue and RNeasy FFPE Kits (Qiagen), respectively, following manufacturer's protocols. Both RNA and DNA were then used to

test for the presence of viruses using a pan-virus microarray (GEO accession number GPL8185) as described before[12]. Lastly a pooled (brain, spleen, liver, heart and lymph node) RNA sample was subjected to whole genome amplification using REPLI-g Cell WGA & WTA kit (Qiagen) as per the manufacturer's instructions before being run on a NextSeq instrument (Illumina) for WGS. The NGS data were analysed by reference-guided, viral reference sequences, and de novo assembly applications of SeqMan NGen 17.3 software (DNASTAR Lasergene) after removing host genome sequences (accession number GCF_000001905.1)[11].

Supplementary Fig. 1 Comparative analysis of the distribution of 25 *Pasteurella* virulence markers in 278 *Pasteurella* genomes and Bisgaard Taxon 45 isolate VF20HR, together with *Pasteurella* species, and source category. The phylogenetic tree was created using core genome MLST. The virulence gene list is based on Peng et al, 2015[13]. Two toxin genes present in the genome of Bisgaard Taxon 45 isolate VF20HR are labelled in red; the PmHAS and NanB proteins have been linked to the pathogenesis of bacterial septicaemia[14]. Source category Animals combines pigs, rabbits, rodents and alpaca; source category Bovine contains cattle, buffalo and bison; source category Ruminants contains all other ruminants; source category Avian is all birds (mostly chickens, turkey and ducks). Virulence factors present are shown in red (for VF20HR) and black (for all other *Pasteurella* genomes). The size bar shows the number of cgMLST alleles different.

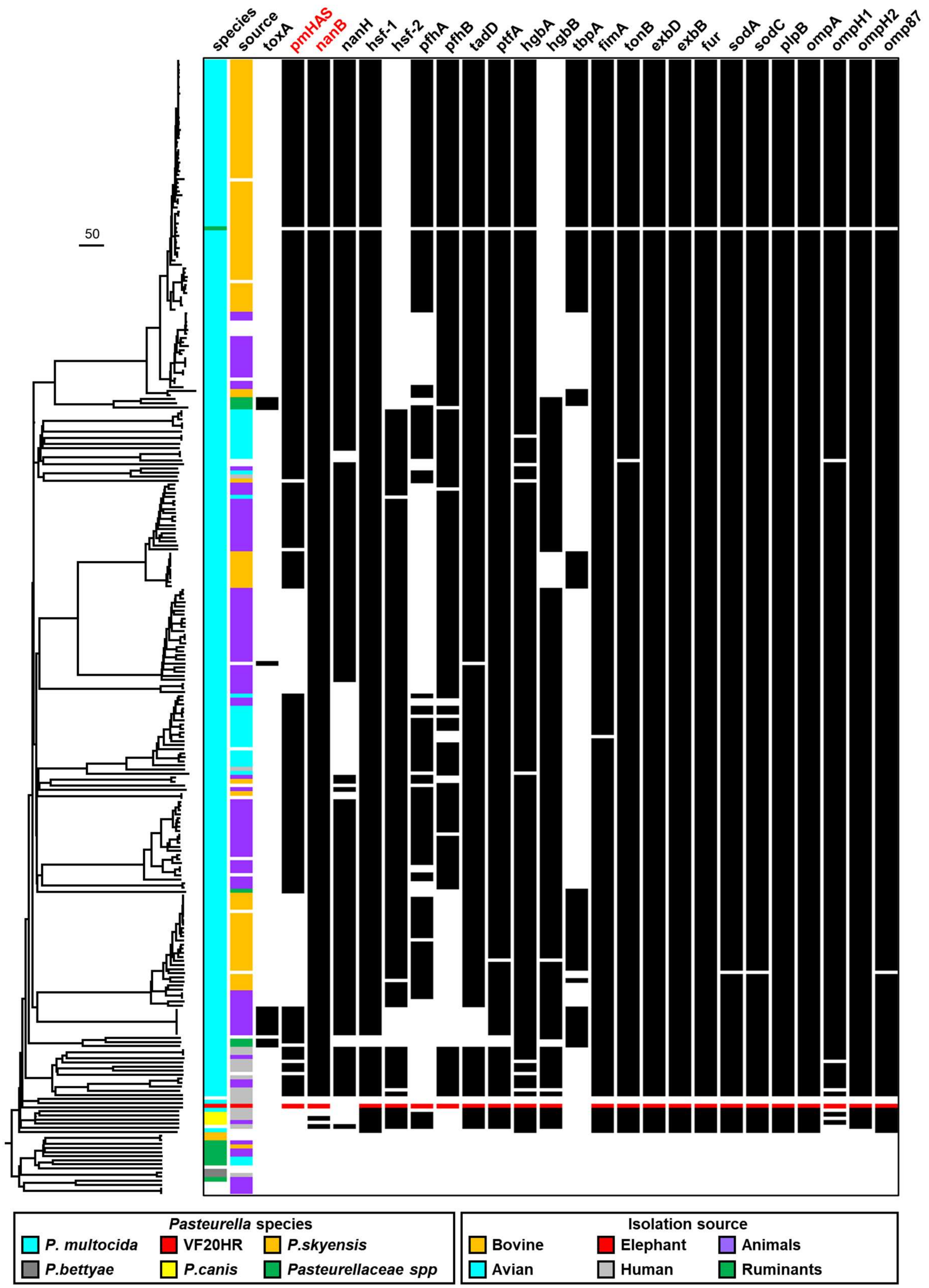

50
species
source
toxA
pmHAS
nanB
nanH
hsf-1
hsf-2
pfhA
pfhB
tadD
ptfA
hgbA
hgbB
tbpA
fimA
tonB
exbD
exbB
fur
sodA
sodC
plpB
ompA
ompH1
ompH2
omp87
Pasteurella species
P. multocida
VF20HR
P.skyensis
P.bettyae
P.canis
Pasteurellaceae spp
Isolation source
Bovine
Elephant
Animals
Avian
Human
Ruminants

Supplementary Tables

Supplementary Table 1: Postmortem and histopathology results for individual elephants

Animal ID	Date Sampled	Sex/ Age (yr)	Necropsy & Histo-pathology Performed	Postmortem Change	Histopathology Findings	Laboratory	Culture	16S rDNA
VF20/112	24/8/2020	M/8	Y	Stage I[a]	Coccobacillary bacterial emboli in brain, spleen; inflammatory changes in spleen, stomach, liver, lung, brain; NSA in oesophagus, heart	VFWT, VetDiagnostix, APHA	*P. multocida*[b] Bisgaard taxon 45[c]	Bisgaard taxon 45
VF20/113	25/8/2020	M/8	Y	Stage I[a]	Coccobacillary bacterial emboli in liver, kidney, spleen; inflammatory changes in liver; NSA in heart	VFWT, VetDiagnostix, APHA	ND	Bisgaard taxon 45
VF20/114	25/8/2020	M/25	N				ND	NA
VF20/115	25/8/2020	M/UNK	N				ND	Bisgaard taxon 45
VF20/116	25/8/2020	M/6	Y	Stage II[a]	Coccobacillary bacterial emboli and inflammatory changes in spleen, lung, kidney, liver	VFWT, VetDiagnostix	ND	Bisgaard taxon 45
VF20/120A	26/8/2020	F/6	N				ND	NA
VF20/120B	26/8/2020	M/18	N				ND	*Clostridium* sp.
VF20/120C	26/8/2020	M/8	N				ND	NA

Animal ID	Date Sampled	Sex/ Age (yr)	Necropsy & Histo-pathology Performed	Postmortem Change	Histopathology Findings	Laboratory	Culture	16S rDNA
VF20/120D	26/8/2020	F/15	N				ND	*Clostridium* sp.
VF20/120E	26/8/2020	F/1.5	N				ND	NA
VF20/124	1/9/2020	M/10	N				ND	*Clostridium vulturis*
VF20/129	7/9/2020	M/18	Y	Stage II[a]	Inflammatory changes in liver, lung; NSA in skeletal muscle; kidney too autolyzed to interpret	VFWT, VetDiagnostix	*P. multocida*[d], *Klebsiella pneumoniae*[c]	Mixed culture signal
VF20/130			N				ND	*P. multocida*
	12/9/2020	M/4	N					Bisgaard taxon 45
VF20/134	16/9/2020	F/18	N				ND	*Clostridium* sp.
VF20/170	9/11/2020	M/30	Y	Stage II[a]	Bacterial colonies in spleen, liver, lung; inflammatory changes in spleen, liver; NSA in kidney	VFWT	No *Pasteurella* spp.[b]	Bisgaard taxon 45

Abbreviations: NSA = no significant abnormalities, VFWT = Victoria Falls Wildlife Trust Laboratory, APHA = Animal and Plant Health Agency Laboratory, ND = not done, NA = no amplification

a = Stage I decomposition represents a carcass with normal appearance in which rigour mortis has set in and blowfly eggs are present; stage II decomposition represents a carcass estimated to be 6–48 hours postmortem, with abdominal bloating, odour of decomposition, and blowfly larvae present

b = result from ZimVet

c = result from VetDiagnostix

d = result from Zimbabwe Central Veterinary Laboratories

Supplementary Table 2: Post-mortem culture, virology, toxicology, PCR, and sequencing results from elephants found dead in north-western Zimbabwe, 2020

Animal ID	Date Sampled	Sex/ Age (yr)	Sample	Test	Result	Laboratory
VF20/112	24/8/2020	M/8	Swab (liver)	Culture	*P. multocida*	ZimVet
			Swab (brain)	Culture	No *Pasteurella* spp.	ZimVet
			Swab (liver)	Culture	Bisgaard taxon 45	VetDiagnostix
			Stomach fluid	HILIC-MS/MS	No toxins detected	CEFAS, Fera
			DNA (liver) – pooled with VF20/113	WGS	Bisgaard taxon 45, no viruses detected	APHA
			FTA card	PCR/sequencing	NA	Design Biologix/Inqaba
			FTA card (spleen)	PCR sequencing	NA	Design Biologix/Inqaba
			FTA card (brain)	PCR sequencing	Bisgaard taxon 45	Design Biologix/Inqaba
VF20/113	25/8/2020	M/8	Stomach fluid	HILIC-MS/MS	No toxins detected	CEFAS, Fera
			DNA (spleen) – pooled with VF20/112	WGS	Bisgaard taxon 45, no viruses detected	APHA
			FTA card (spleen)	PCR/sequencing	Bisgaard taxon 45	Design Biologix/Inqaba
			FTA card (lymph nodes)	PCR/sequencing	Bisgaard taxon 45	Design Biologix/Inqaba
VF20/114	25/8/2020	M/25	FTA card	PCR/sequencing	NA	Design Biologix/Inqaba
VF20/115	25/8/2020	M/UNK	FTA card	PCR/sequencing	Bisgaard taxon 45*	Design Biologix/Inqaba
VF20/116	25/8/2020	M/6	FTA card (spleen)	PCR/sequencing	Bisgaard taxon 45	Design Biologix/Inqaba
			FTA card (tongue)	PCR/sequencing	Bisgaard taxon 45	Design Biologix/Inqaba
			FTA card (blood)	PCR/sequencing	Bisgaard taxon 45	Design Biologix/Inqaba
VF20/120A	26/8/2020	F/6	FTA card	PCR/sequencing	NA	Design Biologix/Inqaba
VF20/120B	26/8/2020	M/18	FTA card	PCR/sequencing	*Clostridium* sp.*	Design Biologix/Inqaba
VF20/120C	26/8/2020	M/8	FTA card	PCR/sequencing	NA	Design Biologix/Inqaba
VF20/120D	26/8/2020	F/15	FTA card	PCR/sequencing	*Clostridium* sp.	Design Biologix/Inqaba
VF20/120E	26/8/2020	F/1.5				
VF20/124	1/9/2020	M/10	FTA card	PCR/sequencing	*Clostridium vulturis**	Design Biologix/Inqaba
VF20/129	7/9/2020	M/18	Blood	Culture	*P. multocida*	Zimbabwe CVL
			Swab (lung)	Culture	No *Pasteurella* spp., *Klebsiella pneumoniae*[c]	VetDiagnostix
			FTA card (liver)	PCR/sequencing	NA	Design Biologix/Inqaba
			FTA card (blood)	PCR/sequencing	Mixed culture signal	Design Biologix/Inqaba
			FTA card (spleen)	PCR/sequencing	NA	Design Biologix/Inqaba
			FTA card (lung)	PCR/sequencing	NA	Design Biologix/Inqaba

Animal ID	Date Sampled	Sex/ Age (yr)	Sample	Test	Result	Laboratory
VF20/130	12/9/2020	M/4	FTA card	PCR/sequencing	*P.multocida**	Design Biologix/Inqaba
			FTA card (blood)	PCR/sequencing	Bisgaard taxon 45	Design Biologix/Inqaba
VF20/134	16/9/2020	F/18	FTA card	PCR/sequencing	Uncultured *Clostridium* sp.	Design Biologix/Inqaba
VF20/170	9/11/2020	M/30	Swab (spleen)	Culture	No *Pasteurella* spp.	ZimVet
			Swab (pus)	Culture	No *Pasteurella* spp.	ZimVet
			Swab (septic wounds)	Culture	No *Pasteurella* spp.	ZimVet
			FTA card (kidney)	PCR/sequencing	NA	Design Biologix/Inqaba
			FTA card (spleen)	PCR/sequencing	Bisgaard taxon 45*	Design Biologix/Inqaba
			FTA card (liver)	PCR/sequencing	Bisgaard taxon 45	Design Biologix/Inqaba

* = mixed culture signal
Abbreviations: HILIC-MS/MS = hydrophilic interaction liquid chromatography with tandem mass spectrometry, WGS = whole genome sequencing, NA = no amplification, CEFAS = Centre for Environment Fisheries and Aquaculture Science, CVL = Central Veterinary Laboratories, APHA = Animal and Plant Health Agency Laboratory
Samples of water and sediment from a pan were also sent to APHA for testing

Supplementary Table 3: Histopathology results from prior elephant deaths, some suggestive of bacterial septicaemia

Animal ID	Date Sampled	Sex/Est. Age (yr)	History relevant to possible cause of death	Blood Smear	Postmortem Samples	Histopathology Findings
VF19/151A	15/08/2019	M/16	No trauma	Y	N	N/A
VF19/151B	18/08/2019	F/30	Hit by train	Y	N	N/A
VF19/151C	11/09/2019	M/20	Died on road, no struggle	Y	Y	Liver: severe inflammatory changes associated with bacterial emboli; lung: severe alveolar oedema; muscle: putrefactive bacteria; NSA in intestine, unspecified gland
VF19/151D	11/09/2019	F/50	Very old, abscess on ear, intestines fluid-filled and ruptured	Y	Y	Lung: slight oedema; spleen and liver: iron pigment accumulation; heart: lipofuchsin pigment present
VF19/151E	19/09/2019	M/8	Very poor condition	Y	Y	Muscle: NSA
VF19/151F	19/09/2019	F/40	Dropped dead on sternum	Y	N	N/A
VF19/151G	25/09/2019	F/8-10	Unknown	Y	Y	Spleen: severe congestion; NSA in heart, lung, salivary gland
VF19/151H	27/09/2019	M/20	Unknown	Y	N	N/A
VF19/151I	27/09/2019	F/10	Unknown	Y	N	N/A
VF19/165A	04/10/2019	F/8-10	Unknown	Y	Y	Spleen: acute bacterial septicaemia
VF19/165B	15/10/2019	M/6-8	Unknown	Y	Y	Liver, spleen: acute bacterial septicaemia
VF19/165C	02/10/2019	F/20	Unknown	Y	N	N/A
VF19/165D	30/09/2019	M10-12	Very thin	Y	Y	Too decomposed for analysis
VF19/165E	11/10/2019	F/4	Large blisters on skin	Y	Y	Too decomposed for analysis

Abbreviations: NSA = no significant abnormalities

Supplementary Notes

Additional results

Gross pathology

Elephants were in average body condition with moderate abdominal, renal, and epicardial fat stores; none were emaciated. The stomach contents were variable, from normal to decreased in volume but very fluid, and the gastric mucosa was congested. The most prominent gross-pathological findings were hepatomegaly and splenomegaly. Although haemorrhages were present, they varied in intensity and distribution (Fig. 2); across the epicardium, liver, lungs, intestinal serosae, hepatic and splenic lymph nodes, and in one case, the diaphragm. The abdomen contained blood-tinged, slightly turbid ascitic fluid. The hepatic serosal surface exhibited mild fibrinopurulent inflammation, and the liver parenchyma was congested with a mottled, haemorrhagic appearance. The lungs showed patchy congestion with mild consolidation. In one elephant, the lungs had generalized consolidation with multiple, pale, firm foci up to 3 cm in diameter with oedematous froth exuding from the bronchi. The one brain and meninges showed intense, diffuse congestion.

Histopathology

The livers of all five elephant carcasses examined exhibited multifocal necrosis with contraction or complete loss of hepatocytes and replacement with intense heterophilic infiltration, forming micro-abscesses. Many of the heterophils were degenerate. The necrotic foci were frequently (>90%) associated with Gram-negative, short coccobacillary bacteria, although there were similar bacterial colonies elsewhere in the liver, in the form of bacterial emboli, with no inflammatory response. This was the most consistent lesion throughout the series. Gram-positive putrefactive bacterial rods were more diffusely present, particularly in livers from carcasses in a more advanced state of decomposition. There was occasional limited bridging fibrosis between portal tracts.

Spleens exhibited congestion of red pulp (n = 2/4) with variable changes in the lymphoid tissue, including depletion or moderate hyperplasia; multifocal heterophil infiltration was present and

associated with colonies of Gram-negative, coccobacillary bacteria. Bacterial aggregates or emboli were present without any associated heterophilic reaction. Histopathological changes in lymph nodes with gross pathologic lesions were similar to those observed in the spleen.

Lung pathology varied among elephants, although diffuse congestion was common (n = 3/4). In some cases, only autolytic changes were present or alveolar oedema with small numbers of heterophils present throughout alveoli. One elephant, which had focal areas of consolidation noted on gross examination, had intense heterophilic invasion of alveolar spaces with obliteration of the normal architecture and replacement with heterophils, macrophages and fibrin, with caseous progression. Incomplete fibrous encapsulation of these foci was noted. Colonies of densely packed Gram-negative coccobacillary bacteria were present in the reactive areas. Elsewhere, alveolae were filled with fibrin-laden fluid and more diffuse heterophils and macrophages or were unaffected. Thromboses of small veins was observed, with or without colonies of coccobacillary bacteria.

None of the kidneys showed significant inflammation, but coccobacillary bacterial emboli were present in all kidneys examined (n = 3/3), predominantly in glomeruli, although without any inflammatory response. Putrefactive bacterial rods were also present and consistent with any autolytic changes.

The one brain showed minimal degenerative, inflammatory changes. There was expansion of the perivascular spaces and many blood vessels contained dense colonies of the bacteria described above, which were mostly adherent to the endothelium and partially occluding the vessels, thus forming bacterial emboli.

Further analysis at APHA using samples from elephants VF20/112 & 113 confirmed these results. Lesions in both elephants were similar and consisted of acute multifocal heterophilic and necrotizing inflammation in liver, spleen, and lymph node, with presence of intralesional Gram-negative bacterial colonies of coccobacillary morphology (Fig. 3). Specifically, elephant VF20/112 displayed necrotizing lesions in spleen and liver, with the additional presence of predominantly heterophilic and fibrinous emboli with Gram-negative coccobacilli (septic emboli) in the pulmonary

vasculature. Presence of Gram-negative bacterial colonies without associated morphological changes was observed in veins and capillaries, prominently in the encephalon. Acute multifocal heterophilic and necrotizing lymphadenitis, hepatitis and splenitis with intralesional Gram-negative coccobacilli was observed in elephant VF20/113.

Most blood smears (n = 13/15) stained with Giemsa contained small to moderate numbers of bacteria with a bipolar, short-rod, or coccobacilli morphology (0.5 - 2μm); Table 1. Blood smears from a single elephant showed *Anaplasma*-like haemoparasites in >50% of erythrocytes. No bacterial rods indicative of *B. anthracis* were present in any blood smears, although putrefactive bacterial rods were present in smears taken from the more decomposed carcasses. In summary the pathological findings were consistent with bacterial septicaemia and bacteraemia at the time of death.

Toxicological analysis

Cyanide was not detected in any stomach contents tested (n = 3) and the targeted toxin analysis that included the analysis saxitoxin analogues (STXs), Tetrodotoxin analogues (TTX) together with anatoxin-a (ATX) and cylindrospermopsin (CYN) provided no positive result in the samples (stomach or waterhole) analysed. While the non-targeted toxicological analysis provided a range of results, no result was outstanding in relation to the clinical picture. The findings by GC-QTOF-MS in the stomach included compounds related to oils, considered non-toxic or the tentative detection of sulphur compounds in the water samples. The most important finding here could be the detection of benzaldehyde in the stomach samples. Benzaldehyde occurs naturally in many plants but is also a degradation product for some cyanogenic glycosides. The LC-TOF-MS identified 5 pesticides and 10 mycotoxins in both stomach samples. Furthermore, cytochalasin J, a fungal metabolite, and triacetin were tentatively identified in the water samples. In relation of the benzaldehyde detection by GC-QTOF-MS, the LC- TOF-MS data was further mined for the presence of mandelonitrile, a component and breakdown product of cyanogenic glycosides, which was tentatively identified in the stomach contents at low levels.

Supplementary References

1. Ryu, C., Lee, K., Yoo, C., Seong, W. K. & Oh, H.-B. Sensitive and rapid quantitative detection of anthrax spores isolated from soil samples by real-time PCR. *Microbiol Immunol* **47**, 693–699 (2003).
2. Kholilullah, Z. A. *et al.* Identification of virulence of *Bacillus anthracis* from soil by multiplex PCR technique in the South Sulawesi province of Indonesia. *Am J Infect Dis Microbiol* **4**, 118–122 (2016).
3. Turner, A. D., Waack, J., Lewis, A., Edwards, C. & Lawton, L. Development and single-laboratory validation of a UHPLC-MS/MS method for quantitation of microcystins and nodularin in natural water, cyanobacteria, shellfish and algal supplement tablet powders. *Journal of Chromatography B* **1074–1075**, 111–123 (2018).
4. Turner, A. D., Boundy, M. J. & Rapkova, M. D. Development and single-laboratory validation of a liquid chromatography tandem mass spectrometry method for quantitation of Tetrodotoxin in mussels and oysters. *J AOAC Int* **100**, 1469–1482 (2017).
5. Turner, A. D. *et al.* Ultrahigh-performance hydrophilic interaction liquid chromatography with tandem mass spectrometry method for the determination of paralytic shellfish toxins and tetrodotoxin in mussels, oysters, clams, cockles, and scallops: collaborative study. *J AOAC Int* **103**, 533–562 (2020).
6. Boundy, M. J., Selwood, A. I., Harwood, D. T., McNabb, P. S. & Turner, A. D. Development of a sensitive and selective liquid chromatography–mass spectrometry method for high throughput analysis of paralytic shellfish toxins using graphitised carbon solid phase extraction. *J Chromatogr A* **1387**, 1–12 (2015).
7. Turner, A. D., McNabb, P. S., Harwood, D. T., Selwood, A. I. & Boundy, M. J. Single-laboratory validation of a multitoxin ultra-performance LC-hydrophilic interaction LC-MS/MS method for quantitation of paralytic shellfish toxins in bivalve shellfish. *J AOAC Int* **98**, 609–621 (2015).

8. Silva, M. *et al.* chewBBACA: A complete suite for gene-by-gene schema creation and strain identification. *Microb Genom* **4**, (2018).

9. Hyatt, D. *et al.* Prodigal: prokaryotic gene recognition and translation initiation site identification. *BMC Bioinformatics* **11**, 119 (2010).

10. Zhou, Z. *et al.* GrapeTree: visualization of core genomic relationships among 100,000 bacterial pathogens. *Genome Res* **28**, 1395–1404 (2018).

11. Dastjerdi, A. *et al.* Novel arterivirus associated with outbreak of fatal encephalitis in European hedgehogs, England, 2019. *Emerg Infect Dis* **27**, 578–581 (2021).

12. Dastjerdi, A., Fooks, A. R. & Johnson, N. Oligonucleotide microarray: applications for Lyssavirus speciation. in *Current Laboratory Techniques in Rabies Diagnosis, Research and Prevention* (eds. Rupprecht, C. & Nagarajan, T.) vol. 1 193–2041 (Elsevier, 2014).

13. Peng, Z. *et al.* *Pasteurella multocida*: genotypes and genomics. *Microbiol Mol Biol Rev* **83**, e00014-19 (2019).

14. Carter, G. R. & Chengappa, M. M. Hyaluronidase production by type B *Pasteurella multocida* from cases of hemorrhagic septicemia. *J Clin Microbiol* **11**, 94–96 (1980).
