# Supplementary material for: Pasteurella sp. associated with fatal septicaemia in six African elephants
Source: Nat Commun. 2023 Oct 25;14:6398. doi: 10.1038/s41467-023-41987-z (PMC10600241; doi:10.1038/s41467-023-41987-z)
Supplement: Supplementary file 3 — Description of additional supplementary files [file 41467_2023_41987_MOESM3_ESM.pdf]

Description of Additional Supplementary Files Document

Supplementary Dataset 1 - A list of the Pasteurella genomes used in the study
