# Supplementary material for: Pasteurella sp. associated with fatal septicaemia in six African elephants
Source: Nat Commun. 2023 Oct 25;14:6398. doi: 10.1038/s41467-023-41987-z (PMC10600241; doi:10.1038/s41467-023-41987-z)
Supplement: Supplementary file 5 — Reporting Summary [file 41467_2023_41987_MOESM5_ESM.pdf]

nature portfolio

Corresponding author(s): Laura Rosen

Last updated by author(s): Sep 14, 2023

Reporting Summary

Nature Portfolio wishes to improve the reproducibility of the work that we publish. This form provides structure for consistency and transparency in reporting. For further information on Nature Portfolio policies, see our Editorial Policies and the Editorial Policy Checklist.

Statistics

For all statistical analyses, confirm that the following items are present in the figure legend, table legend, main text, or Methods section.

n/a	Confirmed	
☒	☐	The exact sample size (*n*) for each experimental group/condition, given as a discrete number and unit of measurement
☒	☐	A statement on whether measurements were taken from distinct samples or whether the same sample was measured repeatedly
☒	☐	The statistical test(s) used AND whether they are one- or two-sided<br>*Only common tests should be described solely by name; describe more complex techniques in the Methods section.*
☒	☐	A description of all covariates tested
☒	☐	A description of any assumptions or corrections, such as tests of normality and adjustment for multiple comparisons
☒	☐	A full description of the statistical parameters including central tendency (e.g. means) or other basic estimates (e.g. regression coefficient) AND variation (e.g. standard deviation) or associated estimates of uncertainty (e.g. confidence intervals)
☒	☐	For null hypothesis testing, the test statistic (e.g. *F*, *t*, *r*) with confidence intervals, effect sizes, degrees of freedom and *P* value noted<br>*Give P values as exact values whenever suitable.*
☒	☐	For Bayesian analysis, information on the choice of priors and Markov chain Monte Carlo settings
☒	☐	For hierarchical and complex designs, identification of the appropriate level for tests and full reporting of outcomes
☒	☐	Estimates of effect sizes (e.g. Cohen's *d*, Pearson's *r*), indicating how they were calculated

Our web collection on statistics for biologists contains articles on many of the points above.

Software and code

Policy information about availability of computer code

Data collection	Postmortem data was compiled in Microsoft Excel 365.
Data analysis	Whole genome sequencing was carried out using MEGA 7.0,. Bowtie2 2.4.2, Samtools 1.12, Shovill 1.1.0, Spades 1.13.3., Prokka 1.14, chewBBACA 2.8.5, Prodigal 2.6.3, GrapeTree 1.5.0. Toxicological analysis was carried out suing MassHunter Unknowns Analysis B.0.9.00 and MassHunter Qualitative 10.0 software. Genetic analysis for viruses was carried out using SeqMan NGen 17.3. Details for analyses are provided in the Methods and Supplemental Methods.

For manuscripts utilizing custom algorithms or software that are central to the research but not yet described in published literature, software must be made available to editors and reviewers. We strongly encourage code deposition in a community repository (e.g. GitHub). See the Nature Portfolio guidelines for submitting code & software for further information.

Data

Policy information about availability of data

All manuscripts must include a data availability statement. This statement should provide the following information, where applicable:

- Accession codes, unique identifiers, or web links for publicly available datasets
- A description of any restrictions on data availability
- For clinical datasets or third party data, please ensure that the statement adheres to our policy

The Illumina sequencing reads and genome assembly of Bisgaard Taxon 45 isolate VF20HR generated in this study have been deposited in the SRA and NCBI

Genome repositories, respectively, and are available fromunder SRA accession code SRR22922729 [https://www.ncbi.nlm.nih.gov/sra/SRR22922729/]), Bioproject accession code PRJNA914783 (accession number SRR22922729) [https://www.ncbi.nlm.nih.gov/bioproject/?term=PRJNA914783], Biosample accession code SAMN32358138 [https://www.ncbi.nlm.nih.gov/biosample/?term=SAMN32358138] and genomeGenBank accession number JAQAHH000000000 [https://www.ncbi.nlm.nih.gov/nuccore/JAQAHH000000000]. The Pasteurella cgMLST scheme is available via Figshare (https://doi.org/10.6084/m9.figshare.21791843.v1).

Human research participants

Policy information about studies involving human research participants and Sex and Gender in Research.

Reporting on sex and gender	No human participants in this study
Population characteristics	No human participants in this study
Recruitment	No human participants in this study
Ethics oversight	No human participants in this study

Note that full information on the approval of the study protocol must also be provided in the manuscript.

Field-specific reporting

Please select the one below that is the best fit for your research. If you are not sure, read the appropriate sections before making your selection.

☒ Life sciences ☐ Behavioural & social sciences ☐ Ecological, evolutionary & environmental sciences

For a reference copy of the document with all sections, see nature.com/documents/nr-reporting-summary-flat.pdf

Life sciences study design

All studies must disclose on these points even when the disclosure is negative.

Sample size	Data were collected opportunistically during a mortality event and the study does not contain experimental data, so no sample size was calculated
Data exclusions	No data were excluded from analysis
Replication	This was not an in vitro experimental study, but the histopathological findings observed at the laboratory in Zimbabwe were independently confirmed in samples sent to laboratories in South Africa and the UK. These groups also independently identified Bisgaard taxon 45, by either 16S rDNA or whole genome sequencing.
Randomization	Randomization was not performed as there was no experimental component to this study
Blinding	Blinding is not relevant to this study as no experimental treatments were implemented

Reporting for specific materials, systems and methods

We require information from authors about some types of materials, experimental systems and methods used in many studies. Here, indicate whether each material, system or method listed is relevant to your study. If you are not sure if a list item applies to your research, read the appropriate section before selecting a response.

Materials & experimental systems

n/a	Involved in the study	
☒	☐	Antibodies
☒	☐	Eukaryotic cell lines
☒	☐	Palaeontology and archaeology
☐	☒	Animals and other organisms
☒	☐	Clinical data
☒	☐	Dual use research of concern

Methods

n/a	Involved in the study	
☒	☐	ChIP-seq
☒	☐	Flow cytometry
☒	☐	MRI-based neuroimaging

Animals and other research organisms

Policy information about studies involving animals; ARRIVE guidelines recommended for reporting animal research, and Sex and Gender in Research

Laboratory animals	This study did not involve laboratory animals.
Wild animals	This study involved 35 wild African elephants, all of which were deceased at the time of examination. No animals were killed as part of this study, all animals were found dead opportunistically. Samples were collected by Victoria Falls Wildlife Trust on behalf of Zimbabwe Parks and Wildlife Management Authority. There is a Memorandum of Understanding between these groups which allows VFWT to conduct wildlife veterinary sampling in the region.
Reporting on sex	Sex was not part of the study design as we collected samples opportunistically by nature of the event. We collected data on sex where possible, and counted 16 males and 9 females. Sex could not be determined in the other animals due to decomposition or if the carcass was only identified from aerial surveys. Given the overall low sample size, no sex-based analyses were performed.
Field-collected samples	All samples were collected from the field, but only from postmortem examinations of animals that had been found dead.
Ethics oversight	This study did not require ethics approval as no live animals were involved.

Note that full information on the approval of the study protocol must also be provided in the manuscript.
